# Sex Differences in Outcomes After Elective and Acute Aortic Surgery—A Single-Centre Experience over the Last Two Decades

**DOI:** 10.3390/jcm13216313

**Published:** 2024-10-22

**Authors:** Magnus Strypet, Caitlin Bozic, Floor J. Mansvelder, Jennifer S. Breel, Evert K. Jansen, Eline S. de Klerk, Desiré E. López González, Henning Hermanns, Susanne Eberl

**Affiliations:** 1 Department of Anaesthesiology, Amsterdam UMC, 1105 AZ Amsterdam, The Netherlands; m.strypet@amsterdamumc.nl (M.S.);; 2 Department of Cardiac Surgery, Amsterdam UMC, 1105 AZ Amsterdam, The Netherlands

**Keywords:** thoracic aortic aneurysm, acute aortic dissection, postoperative complications, aortic surgery, cardiac surgery, postoperative mortality, long-term

## Abstract

**Background/Objectives:** Recent studies show conflicting results regarding sex-related differences in outcomes following aortic surgery. This study primarily evaluated 20-year mortality rates, along with 30-day and one- and five-year rates, and postoperative complications in female and male patients who underwent elective and acute ascending aortic surgery at a tertiary care centre. **Methods:** This retrospective observational study analysed data from 676 adult patients who underwent ascending aortic surgery in Amsterdam UMC, between January 2001 and December 2020. Descriptive statistics, Fisher’s Exact test, Kaplan–Meier survival analysis, and logistic regression were used to assess differences in mortality rates. **Results:** Patient characteristics for elective and acute procedures were different. Females were generally older (elective: 69 vs. 62 years, *p* < 0.001, acute: 70 vs. 62 years, *p* = 0.002), with smaller body surface area (elective/acute: 1.8 vs. 2.1 m^2^, *p* < 0.001), lower preoperative haemoglobin (elective: 8.3 vs. 8.9 mmol/L, *p* < 0.001, acute: 7.8 vs. 8.4 mmol/L, *p* < 0.001) and estimated glomerular filtration rate (elective: 64 vs. 91 mL/min, *p* < 0.001; acute: 67 vs. 83 mL/min, *p* = 0.004). Females undergoing elective procedures had a lower body mass index (25.0 vs. 26.6 kg/m^2^, *p* = 0.006), better left ventricle function (*p* = 0.048) and higher incidence of chronic obstructive pulmonary disease (15 vs. 8%, *p* = 0.032). In elective procedures, the 20-year mortality rate (49% vs. 30%, *p* < 0.001) was significantly higher in females, especially those under 60 years (OR of 3.158 [1.2–8.6], *p* = 0.024). Mortality rates up to one year were comparable. Five-year mortality rate (26% vs. 16%, *p* = 0.027) was higher. Females had longer ventilation times (32% vs. 15%, *p* < 0.001) and intensive care unit stays (2 vs. 1 days, *p* = 0.005). For acute procedures, morbidity and mortality rates were comparable between sexes. **Conclusions:** Females under 60, undergoing elective ascending aortic surgery showed higher long-term all-cause mortality rates. Implementing sex-specific management strategies and extended follow-up could be essential for improving outcomes in this group.

## 1. Introduction

Open surgery on the ascending aorta remains a crucial treatment for aortic pathologies, including aneurysms and dissections. Over recent decades, significant advancements in surgical techniques, perioperative care, and postoperative management have led to improved outcomes, with promising trends in long-term survival rates after thoracic aortic surgery [1,2,3].

Despite these overall improvements, research has uncovered significant sex-specific differences in outcomes after surgery on the ascending aorta. Female patients typically present with a distinct clinical profile, characterised by older age, more comorbidities, and often a progressively aggressive disease course, with higher aortic diameter growth rates [1,3]. Furthermore, females generally undergo surgery at larger aortic diameters relative to body size, compared to males, potentially influencing surgical outcomes [1,3,4].

These sex-related disparities extend beyond preoperative characteristics, affecting both perioperative and long-term outcomes. Several studies have reported higher unadjusted in-hospital mortality rates for females undergoing aortic surgery [4]. Postoperative morbidity was elevated in female patients, with longer intensive care unit stays, extended intubation times, and a higher incidence of specific complications [5,6]. However, the literature presents conflicting results, likely due to variations in study populations, surgical techniques, and urgency of the procedure.

Recent investigations into sex-related differences in intermediate and long-term outcomes following aortic surgery have yielded inconsistent findings [1,6]. While some researchers have found comparable outcomes between sexes, others have demonstrated worse prognoses in females. These contradictions highlight the need for further investigation into this important aspect of ascending aortic surgery. To address these inconsistencies and gain a more comprehensive understanding of sex-related disparities in outcomes, our study primarily aims to evaluate long-term (20-year) mortality rates, as well as short-term (30-day), and intermediate term (one- and five-year) rates, between female and male patients who underwent elective and acute ascending aortic surgery in our centre during the last two decades. By analysing a large cohort of patients, we seek to provide valuable insights into the impact of sex on surgical outcomes across various time points.

## 2. Materials and Methods

### 2.1. Study Design, Setting, and Participants

This retrospective, single-centre, observational study analysed prospectively collected quality assurance data of all adult female and male patients (>18 years) who underwent ascending aortic surgery in the Amsterdam University Medical Centre (Amsterdam UMC), The Netherlands, between January 2001 and December 2020.

### 2.2. Ethics

The Institutional Review Board of Amsterdam UMC confirmed that this study did not fall under the Medical Research involving Human Subjects Act on 10 February 2022 and waived the need for individual patient consent (W22_050 #22.081). This study was registered on Clinical Trials.gov on 10 March 2022 (NCT06554925, last accessed on 15 August 2024). We adhered to the principles outlined in the Declaration of Helsinki (Fortaleza, 2013) [7] and the General Data Protection Regulation [8]. The Strengthening the Reporting of Observational Studies in Epidemiology (STROBE) guidelines were adhered to in the writing of this report [9].

### 2.3. Study Endpoints

The primary endpoint of this study was long term (20 years) all-cause mortality rates in females and males undergoing aortic surgery of the ascending aorta.

Secondary endpoints were the incidence of all-cause mortality in the short and intermediate term (30-day mortality, one- and five-year mortality) and occurrence of postoperative complications between the sexes. Additionally, we planned to conduct subgroup analyses comparing elective versus acute surgery groups, as well as examining age groups divided at 59 years to explore potential menopausal effects, based on findings from Zhu et al. [10].

### 2.4. Definitions and Variables

We used the following definitions.

Ascending aortic surgery:
All surgery on the ascending aorta, including the ascending aorta and aortic arch.
Surgery urgency:

- Elective surgery: admission for surgery.
- Acute surgery: every other form of surgery (emergency, salvage).

Mortality
Mortality rates are all-cause, cumulative over the whole time-period. Cut-off point is the day of death or status on 1 November 2023, whichever came first:

- Thirty-day mortality: day of death within thirty days from day of surgery,
- One-year mortality: day of death within one year from day of surgery,
- Five-year mortality: day of death within five years from day of surgery,
- Twenty-year mortality: day of death within twenty years from day of surgery.

We collected the following variables:

Preoperative parameters: Age (years), body mass index (BMI) (kg/m^2^), body surface area (Du Bois) (BSA) (m^2^), preoperative haemoglobin (mmol/L), estimated glomerular filtration rate (eGFR) (mL/min), left ventricular function (LVF): normal > 50% left ventricular ejection fraction (LVEF), moderately abnormal 30–50% LVEF, severely abnormal < 30% LVEF; NYHA classification (I–IV)

Medical history: Hypertension, atrial fibrillation, hypercholesterolaemia, myocardial infarction, percutaneous transluminal coronary angioplasty (PTCA), extracardiac arteriopathy, chronic obstructive pulmonary disease (COPD), smoking, diabetes, cerebrovascular accident (CVA), transient ischemic attack (TIA), previous heart surgery.

*Postoperative complications*: atrial fibrillation, acute kidney injury (AKI), CVA/TIA. 

Intensive care unit length of stay (ICU LOS), prolonged ventilation ICU (>48 h), mediastinitis, reintervention, tamponade.

For all other definitions, see Appendix A.

### 2.5. Data Sources and Measurements

The data were prospectively collected quality assurance data of patients undergoing surgery of the ascending aorta, which were entered in the mandatory database. Administrative and preoperative data were entered by an administrative secretary. Perioperative clinical and surgical data were collected by the operating surgeon by way of an automated operating room report, filled out on a web-based form on a local server within the firewalls of the hospital. All data were checked after discharge by the same cardiac surgeon (EKJ) over the whole study period. Variables were checked for outliers and crosschecked with the hospital database for their correctness and corrected where necessary. Following surgery, patients received follow-up care from their treating cardiologists at their registered hospitals. To determine all-cause mortality, annual checks of each patient’s mortality status were conducted using the municipal national civil registry, which is linked to social security numbers. The final mortality status check for this study was performed on 1 November 2023.

### 2.6. Sample Size

All patients that underwent aortic surgery in Amsterdam UMC between January 2001 and December 2020 were included in this analysis.

### 2.7. Data Management

Data were checked for accuracy, missing data, outliers, and normality prior to analysis. Normality of continuous data was assessed by visual inspection of histograms, Q-Q plots, and boxplots. Statistical analysis was performed using IBM^®^ SPSS^®^ Statistics 28.0.1.1.

### 2.8. Statistical Analysis

Descriptive statistics were used to present patient characteristics and incidence rates. Variables with non-normal distributions were presented as a median with interquartile range (IQR) and tested with the Mann–Whitney U test. Categorical variables were presented using absolute frequencies and corresponding percentages. Differences in short- and long-term mortality rates between sexes was assessed with Fisher’s Exact test. Mortality rates were analysed using Kaplan–Meier survival curves, with a cut-off point of 59 years, based on a sub-analysis of age and potential effect of menopause as reported by Zhu et al. [10]. A logistic regression was used to determine the association of sex and mortality within the elective and acute surgery groups. A sensitivity analysis was performed on the elective and acute surgery groups. As these groups were different, all results are presented separately. All tests were two-sided, and a *p*-value <0.05 was considered statistically significant.

## 3. Results

### 3.1. Patient Characteristics

**Figure 1:**
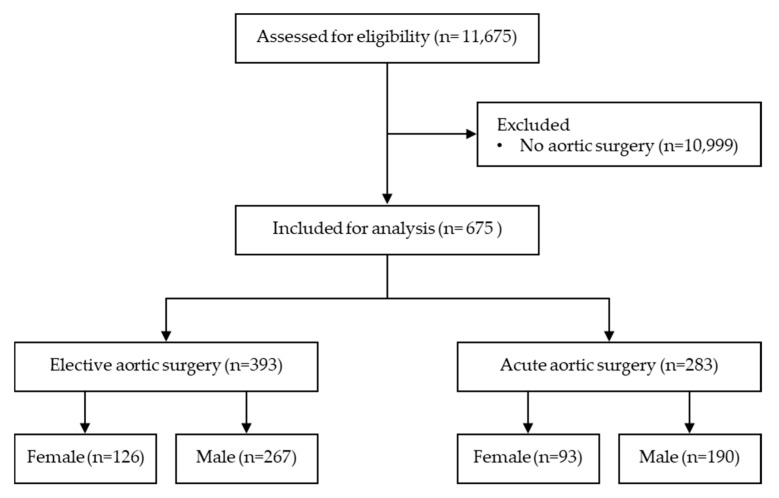
Flow diagram of patients undergoing surgery of the aorta.

Between January 2001 and December 2020, a total of 11,675 patients undergoing cardiac surgery were assessed for eligibility. Out of these, 676 patients underwent aortic surgery and were included in the study cohort (Figure 1). The procedures were categorised as either elective (393 patients, 58%) or acute (283 patients, 42%). Sex distribution within the aortic surgery cohort showed that 219 patients (32%) were female, with 126 (58%) undergoing elective and 93 (42%) undergoing acute procedures. In contrast, 457 patients (68%) were male, with 267 (58%) undergoing elective surgery and 190 (42%) undergoing acute procedures. The median follow-up was 9 (IQR [5–15]) years. Follow-up was available for all patients. Patient characteristics were different between females and males, and between elective and acute patients. No significant difference in surgical urgency was observed between females and males (*p* = 0.868). See Table 1a,b.

Females operated on in an elective setting were significantly older at the time of surgery compared to males (69 [62–74] years vs. 62 [53–70] years, *p* < 0.001). Likewise, females showed a significantly lower BMI (25.0 [22.8–28.7] kg/m^2^ vs. 26.6 [24.2–29.2] kg/m^2^, *p* = 0.006), a smaller BSA (1.8 [1.7–1.9] m^2^ vs. 2.1 [1.9–2.2] m^2^, *p* < 0.001), lower pre-operative haemoglobin levels (8.3 [7.7–8.7] mmol/L vs. 8.9 [8.2–9.4] mmol/L, *p* < 0.001), and a lower eGFR (64 [53–82] mL/min vs. 91 [70–112] mL/min, *p* < 0.001). In addition, the incidence of abnormal LVF (*p* = 0.048) in females was lower but the incidence of COPD (15 vs. 8%, *p* = 0.032) was higher. Other demographic and clinical characteristics were comparable between the sexes. See Table 1a.

Females operated in an acute setting were significantly older at the time of surgery compared to males (70 [58–75] vs 62 [52–71], *p* = 0.002). Likewise, females showed a lower BSA (1.8 [1.7–1.9] 2.1 [1.9–2.2], *p* < 0.001), a lower pre-operative haemoglobin level (7.8 [7.3–8.4] vs. 8.4 [7.6–9.1], *p* < 0.001), and a lower eGFR (67 [51–90] vs. 83 [60–101], *p* = 0.004).

Males, on the other hand, had a greater likelihood of previous heart surgery (0 vs. 5%, *p* = 0.034). Other demographic and clinical characteristics were comparable between the two sexes. See Table 1b.

### 3.2. Primary Outcome

For patients undergoing elective ascending aortic surgery, cumulative mortality rate was significantly higher in females compared to males at 20 years [62 (49%) vs. 80 (30%), *p* < 0.001]. The 20-year mortality rate for acute aortic surgery was comparable between the sexes [45 (48%) vs. 92 (48%), *p* = 1.000] (Table 2).

### 3.3. Secondary Outcomes

Mortality rates in patients undergoing elective surgery at 30 days [8 (6%) vs. 15 (6%), *p* = 0.819] and one year [17 (13%) vs. 26 (10%), *p* = 0.300] were similar between sexes. The five-year [32 (25%) vs. 42 (16%), *p* = 0.027] mortality rate was significantly higher in females compared to males (Table 2a).

Mortality rates in patients undergoing acute surgery were comparable between the sexes at 30 days [21 (23%) vs. 41 (22%), *p* = 0.879], one year [25 (27%) vs. 51 (27%), *p* = 1.000], and five years [31 (33%) vs. 65 (34%), *p* = 1.000] (Table 2b).

After elective procedures, females were ventilated longer in the ICU than males (36 (32%) vs. 36 (15%) *p* < 0.001) and the length of stay was longer (2 [1–7] vs. 1 [1–4] days, *p* = 0.005). There were no further differences in post-operative in-hospital complications between the sexes (Table 3a).

No significant differences in post-operative in-hospital complications after acute aortic surgery were found between the sexes (Table 3b).

### 3.4. Mortality

**Figure 2:**
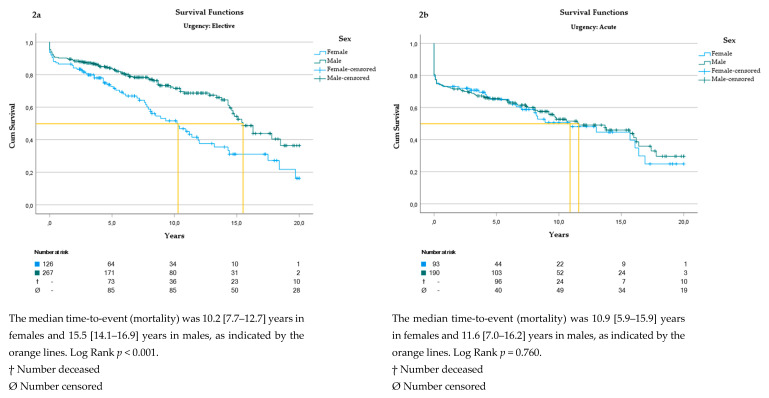
(**a**) Survival analysis in patients undergoing elective aortic surgery. (**b**) Survival analysis in patients undergoing acute aortic surgery.

The Kaplan–Meier curve (Figure 2a,b) revealed a higher mortality rate for females undergoing elective aortic surgery (log rank *p* < 0.001). For acute ascending aortic surgery, there was no statistical difference between the sexes.

**Figure 3:**
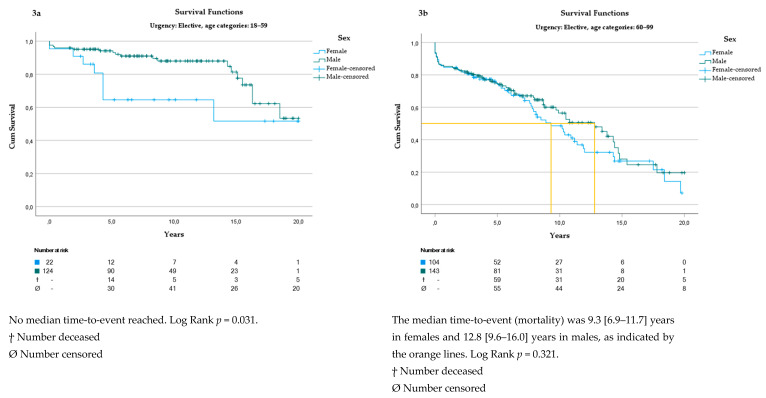
(**a**): Survival analysis in patients 18–59 years undergoing elective aortic surgery. (**b**) Survival analysis in patients over 60 years undergoing elective aortic surgery.

When exploring the effects of age and mortality, female patients younger than 60 years undergoing elective aortic surgery had a significantly higher mortality rate, compared to males in the same age group (*p* = 0.031) (Figure 3a). The unadjusted logistic regression of sex versus mortality showed an OR of 3.158 ([1.2–8.6], *p* = 0.024) for females.

**Figure 4:**
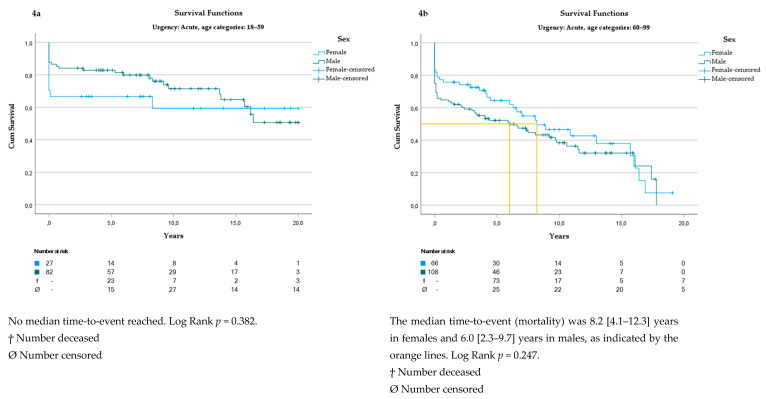
(**a**) Survival analysis in patients 18–59 years undergoing acute aortic surgery. (**b**) Survival analysis in undergoing acute aortic surgery.

There was no difference in mortality rate between patients older than 60 years or undergoing acute aortic surgery (Figure 3b and Figure 4a,b).

## 4. Discussion

Our analysis of patients undergoing ascending aortic surgery over the past two decades at our university centre revealed distinct differences in patient characteristics and mortality rates between elective and acute procedures. Females in the elective group were significantly older at the time of surgery and exhibited lower BMI, BSA, pre-operative haemoglobin levels, and eGFR compared to males. While mortality and complication rates were comparable between sexes in acute surgeries, females undergoing elective surgeries experienced significantly higher 5-year and 20-year mortality rates, particularly in the age group below 60 years. Furthermore, females faced worse postoperative outcomes, including longer ventilation times and extended ICU stays.

### 4.1. Literature in the Field

Our patient population aligns with those reported in the literature on aortic surgery. Consistent with our findings, several studies have shown that female patients undergoing aortic surgery tend to be older and have a higher prevalence of COPD compared to male patients.

Gökalp et al. [11], analysing data from The Netherlands Heart Registry between 2013 and 2017, found that female patients were significantly older (67 vs. 62 years, *p* < 0.001) and had higher rates of COPD (12.3% vs. 9.1%, *p* = 0.011), but fewer PTCAs in their history (3.2% vs. 5.0% *p* = 0.022). Similarly, van Kampen et al. [6], in their single-institution study of 1773 elective patients undergoing proximal aortic surgery (2000–2018), reported longer ventilation times and longer ICU stays for females. These findings mirror our results.

Voigt et al. [3] performed a single-centre study on elective aortic surgery with a 25-year follow-up. They also found females to be significantly older (66 vs. 56 years, *p* < 0.001) with significantly smaller BSA 1.78 vs. 2.04 *p* < 0.001 and correspondingly higher aortic size indices (cm/cm^2^) (3.15 vs. 2.7 cm^2^, *p* < 0.001). Males had more instances of previous cardiac disease or cardiac surgery. While they observed lower (not significant) short-term mortality rate in females (0.9% vs. 2.0%), long-term survival rates were lower, particularly in the 50–65 age group (*p* = 0.020), aligning with our findings.

Regarding short-term mortality rates, our results are consistent with several studies showing no significant differences between sexes. Friedrich et al. [12], in a large retrospective study (n = 905) of elective aortic surgeries between 2001 and 2015, found no statistical difference in 30-day mortality rates between propensity-matched female and male patients (4.9% vs. 3.9%, *p* = 0.480). Similar results were observed in acute dissection cases. Females were older (70 vs. 61 years, *p* < 0.001), but showed no significant difference in 30-day mortality rates (19% vs. 16.5% *p* = 0.545) [13].

Other studies on acute settings, including that of Rylski et al. [14], a meta-analysis by Carbone et al. [15], and a study by Bhatt et al. [16], also reported comparable short-term mortality rates between sexes, despite females being older. However, these two later studies noted better long-term survival in males, possibly due to less adequate follow-up programs for females.

The short-term in-hospital mortality rate reported by Gökalp et al. [11] was higher for females (5.1% vs. 2.7%, *p* = 0.003). The in-hospital mortality rate is not only influenced by patient selection and surgical technique, but also by patient care in the whole chain of care–: cardiology, cardiac anaesthesia, cardiac surgery, and intensive care, and thus has many confounders that could explain different outcomes in different centres.

Interestingly, the study of Preventza et al. [5], a 30-year study of both elective and emergency patients (n = 3745), found a comparable 30-day mortality rate (7.5% vs. 5.6%, *p* = 0.06) but no significant difference in long-term survival at five and ten years after propensity matching of 1153 female/male pairs. However, their combined analysis of elective and emergency surgeries may have masked potential differences in outcomes between these two categories.

### 4.2. Future Perspectives

Our findings contribute to the growing body of evidence suggesting that female patients undergoing aortic surgery tend to be older, have more comorbidities, and higher aortic indices but undergo fewer cardiologic or cardiosurgical interventions compared to their male counterparts. The observed differences in long-term outcomes, particularly in elective cases, warrant further investigation into potential contributing factors and the development of sex-specific management strategies.

Considering that the patients with the highest mortality rates are in the elective surgery group, it should be possible to implement a preventative strategy to lower mortality rates in females. The earlier medical evaluation and treatment of males may contribute to their better outcomes. Thus, the younger females at risk should be identified and evaluated sooner. The higher aortic diameter indices in females, especially those with shorter stature or lower body surface area (BSA), indicate that intervention parameters for aortic surgery should be tailored separately for each sex, as the current guidelines are based on males [17]. This tailoring could potentially address the higher long-term mortality rates observed in females. Implementing sex-specific follow-up and rehabilitation programs could also help bridge this gap. Additionally, investigating patient-reported outcomes to understand the specific issues and challenges faced by females in the postoperative course is crucial for improving their long-term care and quality of life.

### 4.3. Limitations

The results of the present study should be considered in light of several limitations. Firstly, this data analysis is retrospective, which limits the findings to descriptive statistics and associations and introduces the risk of confounding factors. Mortality is described as all-cause mortality. It was therefore not possible to specify the specific reasons for, for instance, the higher mortality in elective aortic surgery in the population of females under 60 years of age. Potentially, there may be missing data due to it not being documented in the patient electronic file. These data were not transferred to the database. Secondly, this is a single-centre study, which may not be generalisable to all populations. Amsterdam UMC is a tertiary care centre, meaning it primarily treats highly complex cases, which may not reflect the broader patient population. Lastly, data were gathered over a 20-year period. While the group of surgeons at our centre has remained largely consistent, surgical skills, techniques, devices, but also cardiopulmonary bypass and anaesthesia techniques and pre- and post-operative care have evolved significantly over these two decades. This may have influence on mortality rates and incidence of postoperative complications.

### 4.4. Strengths

This study possesses several notable strengths that enhance its validity and relevance. Firstly, it included all patients undergoing aortic surgery in a tertiary care centre over a 20-year period, minimizing selection bias and ensuring a representative sample. The study encompassed both elective and acute surgeries, as well as all procedures involving the aorta, providing a comprehensive overview of outcomes relating to aortic surgical outcomes. Documentation was consistently managed by three surgeons, ensuring uniformity in data collection. Additionally, rigorous post-discharge auditing of all data by the same surgeon (EKJ) throughout the study period enhanced accuracy and consistency. The verification of mortality status through the Dutch database for registers of persons (Basisregistratie Personen) on 1 November 2023 ensured complete follow-ups for all patients, effectively eliminating loss to follow-up bias. These meticulous data collection and verification processes resulted in high-quality data with minimal missing or incorrectly entered information. Finally, the median follow-up of 10 years represents a significant strength, allowing for robust analysis of long-term outcomes and trends in patients undergoing aortic surgery.

## 5. Conclusions

This study reveals important sex-related differences in outcomes following ascending aortic surgery. While short-term mortality rates for both acute and elective surgeries are comparable between sexes, females undergoing elective procedures show significantly higher long-term mortality rates, particularly in the age group under 60 years. Based on our data, we cannot fully explain the differences in mortality found in our study between the sexes, but female patients tend to be older, have more comorbidities, and undergo fewer prior cardiac interventions compared to males. These findings underscore the need for sex-specific management strategies in elective aortic surgery, including tailored preoperative evaluation, sex-specific operative techniques, and extended follow-up care. Implementing such targeted approaches could potentially improve long-term outcomes and reduce disparities between female and male patients undergoing elective aortic surgery the ascending aorta.

## Figures and Tables

**Table 1:** (a,b) Characteristics of patients undergoing elective and acute aortic surgery.

(**a**). Patient characteristics—elective surgery				
Characteristic	Total *N* = 393	Female *N* = 126	Male *N* = 267	*p*-value
Age, years	65 [56–72]	69 [62–74]	62 [53–70]	**<0.001**
BMI, kg/m^2^	26.2 [23.9–29.1]	25.0 [22.8–28.7]	26.6 [24.2–29.2]	**0.006**
BSA, m^2^	2.0 [1.8–2.2]	1.8 [1.7–1.9]	2.1 [1.9–2.2]	**<0.001**
Haemoglobin, mmol/L	8.7 [8.0–9.3]	8.3 [7.7–8.7]	8.9 [8.2–9.4]	**<0.001**
eGFR, mL/min	80 [60–105]	64 [53–82]	91 [70–112]	**<0.001**
CVA/TIA	35 (9)	13 (10)	22 (8)	0.570
Extracardiac arteriopathy	106 (27)	33 (26)	73 (27)	0.903
Atrial Fibrillation	61 (16)	18 (14)	43 (16)	0.766
Hypertension	175 (45)	60 (48)	115 (43)	0.447
Left ventricular function				**0.048**
Normal	337 (86)	116 (92)	221 (83)	
Moderately abnormal	41 (10)	7 (6)	34 (13)	
Severely abnormal	15 (4)	3 (2)	12 (4)	
Myocardial infarction	17 (4)	7 (6)	10 (4)	0.432
PTCA	26 (7)	11 (9)	15 (6)	0.278
Previous heart surgery	76 (19)	18 (14)	58 (22)	0.100
NYHA classification				0.320
I	295 (75)	94 (75)	201 (75)	
II	16 (4)	3 (2)	13 (5)	
III	79 (20)	29 (23)	50 (19)	
IV	3 (1)	0 (0)	3 (1)	
COPD	40 (10)	19 (15)	21 (8)	**0.032**
Smoking	56 (14)	22 (17)	34 (13)	0.219
Diabetes	20 (5)	4 (3)	16 (6)	0.327
Hypercholesterolaemia	74 (19)	28 (22)	46 (17)	0.269
(**b**). Patient characteristics—acute surgery				
Characteristic	Total *N* = 283	Female *N* = 93	Male *N* = 190	*p*-value
Age, years	65 [53–72]	70 [58–75]	62 [52–71]	**0.002**
BMI, kg/m^2^	25.5 [23.8–28.1]	25.1 [23.4–28.0]	25.9 [24.2–28.1]	0.128
BSA, m^2^	2.0 [1.8–2.1]	1.8 [1.7–1.9]	2.1 [1.9–2.2]	**<0.001**
Haemoglobin, mmol/L	8.1 [7.5–8.9]	7.8 [7.3–8.4]	8.4 [7.6–9.1]	**<0.001**
eGFR, mL/min	77 [57–98]	67 [51–90]	83 [60–101]	**0.004**
CVA/TIA	25 (9)	8 (9)	17 (9)	1.000
Extracardiac arteriopathy	113 (40)	37 (40)	76 (40)	1.000
Atrial Fibrillation	33 (12)	10 (11)	23 (12)	0.845
Hypertension	93 (33)	29 (31)	64 (34)	0.689
Myocardial infarction	7 (2)	3 (3)	4 (2)	0.687
Left ventricular function				0.458
Normal	257 (91)	86 (93)	171 (90)	
Moderately abnormal	19 (7)	4 (4)	15 (8)	
Severely abnormal	7 (2)	3 (3)	4 (2)	
Previous heart surgery	10 (4)	0 (0)	10 (5)	**0.034**
PTCA	9 (3)	3 (3)	6 (3)	1.000
NYHA classification				0.312
I	226 (80)	73 (78)	153 (81)	
II	1 (0)	1 (1)	0 (0)	
III	32 (12)	9 (10)	23 (12)	
IV	23 (8)	10 (11)	13 (7)	
COPD	19 (7)	7 (8)	12 (6)	0.801
Smoking	28 (10)	6 (6)	22 (12)	0.208
Diabetes	10 (4)	1 (1)	9 (5)	0.174
Hypercholesterolemia	17 (6)	8 (9)	9 (5)	0.285

Data are shown in median and [interquartile range] or numbers (percentage). *p*-values were calculated with Fisher’s exact test or Pearson chi-square where applicable. Statistical significance assumed at *p* < 0.05 and highlighted in bold. Abbreviations: BMI = Body Mass Index; BSA = Body Surface Area; PTCA = Percutaneous Transluminal Coronary Angioplasty; NYHA = New York Heart Association; COPD = Chronic Obstructive Pulmonary Disease; eGFR = Estimated Glomerular Filtration Rate; CVA = Cerebrovascular Accident; TIA = Transient Ischaemic Attack; Pre-op = pre-operative. Missing from elective surgery: BMI *N* = 10 (3%); BSA *N* = 10 (3%); haemoglobin pre-op *N* = 22 (6%); eGFR *N* = 8 (2%). Missing from acute surgery: BMI *N* = 10 (4%); BSA *N* = 10 (4%); haemoglobin pre-op *N* = 23 (8%); eGFR *N* = 6 (2%); atrial fibrillation *N* = 2 (1%); NYHA *N* = 1 (0%).

**Table 2:** (a,b) All-cause mortality rates in patients undergoing elective and acute aortic surgery.

(**a**) Mortality rates in patients undergoing elective aortic surgery.				
	Total *N* = 393	Female *N* = 126	Male *N* = 267	*p*-value
20 years	142 (36)	62 (49)	80 (30)	**<0.001**
30 days	23 (6)	8 (6)	15 (6)	0.819
1 year	43 (11)	17 (13)	26 (10)	0.300
5 years	74 (19)	32 (25)	42 (16)	**0.027**
(**b**) Mortality rates in patients undergoing acute aortic surgery.				
	Total *N* = 283	Female *N* = 93	Male *N* = 190	*p*-value
20 years	137 (48)	45 (48)	92 (48)	1.000
30 days	62 (22)	21 (23)	41 (22)	0.879
1 year	76 (27)	25 (27)	51 (27)	1.000
5 years	96 (34)	31 (33)	65 (34)	1.000

Data are shown as numbers with percentage. Percentages are per column. *p*-values were calculated with Fisher’s Exact test. Statistical significance assumed at *p* < 0.05 and highlighted in bold.

**Table 3:** (a,b) Postoperative, in-hospital complications in patients undergoing elective and acute aortic surgery.

(**a**) In-hospital complications in patients undergoing elective surgery.				
	Total *N* = 393	Female *N* = 126	Male *N* = 267	*p*-value
Acute kidney injury	13 (3)	4 (3)	9 (3)	1.000
Atrial Fibrillation	78 (20)	26 (21)	52 (20)	0.788
CVA/TIA	18 (5)	6 (5)	12 (5)	1.000
Prolonged ventilation ICU (>48 h)	72 (20)	36 (32)	36 (15)	**<0.001**
ICU LOS	1 [1–5]	2 [1–7]	1 [1–4]	**0.005**
Mediastinitis	1 (0)	0 (0)	1 (0)	1.000
Reintervention	46 (12)	19 (15)	27 (10)	0.179
Tamponade	18 (5)	7 (6)	11 (4)	0.606
(**b**) In-hospital complications in patients undergoing acute surgery.				
	Total *N* = 283	Female *N* = 93	Male *N* = 190	*p*-value
Acute kidney injury	36 (13)	8 (10)	28 (17)	0.181
Atrial Fibrillation	61 (22)	21 (23)	40 (21)	0.761
CVA/TIA	31 (11)	10 (11)	21 (11)	1.000
Prolonged ventilation ICU (>48 h)	117 (46)	35 (42)	82 (49)	0.349
ICU LOS	3 [1–15]	3 [1–8]	4 [1–16]	0.200
Mediastinitis	2 (1)	0 (0)	2 (1)	1.000
Reintervention	34 (12)	11 (12)	23 (12)	1.000
Tamponade	19 (7)	7 (8)	12 (6)	0.801

Data are shown as numbers (percentage) or median [interquartile range]. Percentages are per column. p-values were calculated with Fisher’s exact test. Statistical significance assumed at *p* < 0.05 and highlighted in bold. Abbreviations: CVA = Cerebrovascular accident; TIA = Transient ischaemic attack; ICU = Intensive care unit; LOS = Length of stay. Missing from elective aortic surgery: acute kidney injury *N* = 28 (7%); prolonged ventilation ICU *N* = 39 (10%). Missing from acute aortic surgery: acute kidney injury *N* = 34 (12%); prolonged ventilation ICU *N* = 30 (11%).

## Data Availability

The raw/processed data required to reproduce the above findings cannot be shared at this time as the data also forms part of an ongoing study.

## Appendix A. Abbreviations (Translated to English from The Netherlands Heart Registry Handbook v22.2.1, 20 January 2022

**Overview of definitions used in NHR data registration.**

Acute Kidney Injury (AKI): Renal failure is present if one or more of the Society of Thoracic Surgeons (STS) criteria, was present postoperatively:

- Renal replacement therapy (dialysis, Continuous veno-venous haemofiltration (CVVH)) that was not present preoperatively.
- Highest postoperative creatinine value > 177 µmol/L and twice the preoperative value (if preoperative value = the value of the creatinine on which the EuroSCORE was calculated).

Example 1: A preoperative value of 76 µmol/L and a postoperative value of 182 µmol/L means renal failure.

Example 2: A preoperative value of 102 µmol/L and a postoperative value of 178 µmol/L means no renal failure.

Anaemia: Preoperative Hb level of 13.0g/dL or lower for men and 12.0g/dL for females.

Aortic surgery: Surgery on the aorta (ascending aorta, aortic arch, and/or descending aorta).

Creatine level: The most recent preoperative concentration of creatinine in the blood, measured in micromoles per litre (μmol/l), within the last 3 months prior to the current intervention.

Extracardiac arteriopathy: If one or more of the following criteria are present:

- Intermittent Claudication
- Carotid occlusion or >50% stenosis
- Amputation due to arterial disease
- Previous or planned surgery on abdominal aorta, arteries of the limbs or carotids.

Left Ventricle ejection fraction (LVEF): The percentage of the end-systolic volume of blood in the left ventricle relative to the end-diastolic volume, also known as the relative stroke volume. The most recent determination documented in a diagnostic report prior to the intervention was used. The recorded ejection fraction was to be determined no more than 6 months before the intervention.

NYHA-class: The New York Heart Association (NYHA) functional classification for heart failure. The highest class was recorded within the 2 weeks prior to the current intervention; Class I: No dyspnoea with ordinary physical activity; Class II: Mild dyspnoea with ordinary physical activity; Class III: Marked dyspnoea with less than ordinary physical activity; Class IV: Dyspnoea at rest.

Preoperative haemoglobin value: The latest preoperative concentration of haemoglobin in the blood, measured in grams per decilitre (g/dL).

Previous cardiac surgery: Previous cardiac surgery involving the opening of the pericardium.

Previous cerebrovascular accident (CVA): permanent neurological dysfunction diagnosed by a neurologist due to focal ischaemia of brain, spinal cord or retina, caused by acute infarction of the neurological tissue due to thrombosis, embolism, systemic hypoperfusion or haemorrhage.

Reintervention: due to a complication of the current intervention within 30 days. This also includes reinterventions performed after the patient has been discharged from the hospital in question.

This concerns the first reintervention after the initial closure of the thorax. This applies to all causes, except for the opening of the sternum due to mediastinitis or refixation of the sternum.

- Bleeding/Tamponade: Reintervention due to bleeding, tamponade (this also includes subxiphoid relief of a tamponade by the thoracic surgeon). Puncture is also included.
- Cardiac problems: Requiring surgery with or without the use of extracorporeal circulation (ECC). This includes revision of anastomosis, reperfusion, and similar procedures. Also, performing a new procedure during the same admission (for example, a CABG patient who, after a few days, still requires a valve replacement or a new CABG).
- Other: All other causes, except the opening of the sternum for drainage due to mediastinitis (excluding refixation of the sternum, which is recorded under the variable "Sternal refixation within 30 days").

Thoracic aortic surgery: Surgical intervention on the ascending aorta, aortic arch, or descending aorta during the current procedure.
